# The use of scanning electron microscopy and fixation methods to evaluate the interaction of blood with the surfaces of medical devices

**DOI:** 10.1038/s41598-024-55136-z

**Published:** 2024-02-26

**Authors:** Martina Nalezinková, Jan Loskot, Alena Myslivcová Fučíková

**Affiliations:** 1 Department of Biology, Faculty of Science, University of Hradec Králové, Rokitanského 62, Hradec Králové, 500 03 Czech Republic; 2 Department of Physics, Faculty of Science, University of Hradec Králové, Rokitanského 62, Hradec Králové, 500 03 Czech Republic

**Keywords:** Cell adhesion, Medical research, Biomaterials

## Abstract

Testing the hemocompatibility of medical devices after their interaction with blood entails the need to evaluate the activation of blood elements and the degree of their coagulation and adhesion to the device surface. One possible way to achieve this is to use scanning electron microscopy (SEM). The aim was to develop a novel SEM-based method to assess the thrombogenic potential of medical devices and their adhesiveness to blood cells. As a part of this task, also find a convenient procedure of efficient and non-destructive sample fixation for SEM while reducing the use of highly toxic substances and shortening the fixation time. A polymeric surgical mesh was exposed to blood so that blood elements adhered to its surface. Such prepared samples were then chemically fixed for a subsequent SEM measurement; a number of fixation procedures were tested to find the optimal one. The fixation results were evaluated from SEM images, and the degree of blood elements’ adhesion was determined from the images using ImageJ software. The best fixation was achieved with the May–Grünwald solution, which is less toxic than chemicals traditionally used. Moreover, manipulation with highly toxic osmium tetroxide can be avoided in the proposed procedure. A convenient methodology for SEM image analysis has been developed too, enabling to quantitatively evaluate the interaction of blood with the surfaces of various medical devices. Our method replaces the subjective assessment of surface coverage with a better-defined procedure, thus offering more precise and reliable results.

## Introduction

Hemocompatibility testing of medical devices (MD) entails the need to evaluate the activation of blood elements and the rate of coagulation^1^^,^ which can be determined either on the basis of certain specific markers^2,3^ (fibrinopeptide A, thrombin-antithrombin complex, etc.) or by morphological evaluation using microscopy^4^. Scanning electron microscopy (SEM) enables direct assessment of the interaction between the tested MD and blood, the degree of blood cells’ adhesion on the MD surface, and, at the same time, the state of blood cells^5^. A single analysis thus provides a relatively large and complex amount of data that helps to evaluate the thrombogenic potential of a given agent.

To perform a morphological evaluation of blood elements attached to an MD surface, it is necessary to do a proper and very sensitive fixation of such a sample just after the interaction of the blood with the tested MD. The interaction can take place, for example, in the tubes of a Chandler loop, ensuring blood circulation at a constant temperature of 37 °C by means of a heated water bath^6^. All fixation steps are crucial for the subsequent evaluation and to obtain meaningful results. Careful fixation of blood is essential due to its instability. To ensure a reliable assessment of blood interaction with the tested material, appropriately low concentrations of anticoagulant and stabilizing agents have to be used^7^.

Cell fixation ensures the preservation of the original shapes and state of the cells, prevents autolysis, strengthens the cell membranes and thus protects them from further damage during subsequent work with the sample^8^. The key process of fixation is the stabilization of proteins, most often by crosslinking of individual molecules^9^. The fixation time may vary significantly, depending on the fixation substance used, the type of tissue or cells involved, the amount (thickness) of the sample, as well as the temperature. To choose the proper fixative, the rate of diffusion of a particular tissue solution and the fixation method are most important^10^.

There are various procedures to fix cells intended for SEM measurements, but a comprehensive methodology for fixing an MD with blood attached to its surface has not been published yet. In addition, the fixation procedures themselves have a variety of modifications and use different types of chemicals or combinations of solutions with different concentrations. Very often, toxic substances, such as osmium tetroxide (OsO_4_), glutaraldehyde (GA) and others, are used for fixation^11–13^.

Glutaraldehyde frequently serves as a fixative for SEM^14^ thanks to its ability to rapidly kill the cells and crosslink their proteins firmly. It can be used alone or combined with formaldehyde (FA)^15^. Formaldehyde is most often used in the form of a 4% solution and works on the principle of stabilizing proteins by forming crosslinks between lysine residues^16^. On the contrary, GA creates bonds mainly between pyridine residues and has a lesser effect on the morphological properties of cells^17^. It is most frequently used as a 2–5% solution in a phosphate or cacodylate buffer. GA contains two functional groups that enable it to bind amino acids of proteins^11^.

A disadvantage of glutaraldehyde is its toxicity, which manifests itself, e.g., in severe irritation of eyes, nose, throat, and lungs, headaches, drowsiness, and dizziness. Another drawback could be its instability at a higher (even laboratory) temperature, leading to its degradation. For this reason, it is always recommended to work with fresh solutions of a lower concentration and check their quality regularly or to purify the solution before use^18,19^. The GA molecule is also large (100 g/mol) compared with FA (30 g/mol), negatively affecting its penetration rate^20–22^.

FA is used either alone instead of GA or together with it^14,23^. As these substances fix in slightly different ways and have different speeds of penetration through the membrane, paraformaldehyde (PFA) is sometimes preferred for finer applications, such as SEM^24^. Commercially, FA is sold in an aqueous solution with a concentration of around 40% under the name formalin. In this form, however, its fixing capabilities are impaired, as the solution also contains other degradation products and protein stabilizers. For this reason, a fresh 2–5% solution prepared by depolarizing PFA, which does not disrupt its configuration, is more often used for these purposes^11,25,26^.

The advantage of FA is its availability, affordability, and favourable fixation properties, including fast penetration into the sample, especially at a higher temperature. Its main disadvantages are toxicity, carcinogenicity, and low fixation speed^26^.

The fixation with GA and/or FA is usually followed by a further step involving the use of osmium tetroxide, which additionally stabilizes membrane lipids^27^. Osmium tetroxide also crosslinks proteins^28^. Of all substances, it best preserves the physiological structure of cells, but its drawbacks are the slow penetration rate and high toxicity. Other disadvantages include the formation of dense precipitates during the reaction with ethanol subsequently used for the dehydration of the samples. For this reason, washing is a crucial intermediate step, which thoroughly removes the remains of the fixative solution^11^.

For electron microscopy, OsO_4_ is usually used at a concentration of 1–2%. It can be prepared as an aqueous solution or dissolved in the same buffer used to prepare the primary fixative^27^. The osmium tetroxide molecule is relatively large (254 g/mol), leading to a lower penetration rate^27,29^. The rate of fixation can be increased by a higher temperature^30^. For SEM, the essential property of osmium is its ability to strengthen surfaces and, thanks to its high atomic number, to reduce the risk of sample charging, thus providing a clearer image^31^.

In some cases, combinations of several types of substances are used for primary fixation and subsequent postfixation. The method most commonly used in electron microscopy is aldehyde fixation and subsequent postfixation with osmium tetroxide or 10% formalin^32^.

Since the cells themselves contain a lot of water and at the same time the fixatives are mostly water-based, the samples need to be dehydrated after their fixation. For this purpose, alcohols or acetone are commonly used. Their main advantage is fast penetration and preservation of enzymatic and immunological activity when used at low temperatures^32,33^. Typically, the sample is first soaked in an aqueous alcohol solution and transferred at set intervals with progressively more concentrated solutions, until no water remains in the sample^34^. Dehydration is a crucial step for subsequent drying before exposing the sample to a vacuum in an SEM^35^.

Samples finally transferred to absolute ethanol can then be carefully completely dried using the critical point drying method (CPD) and coated with a thin metal layer^13,35,36^. Gold, palladium, or platinum are most commonly used^37,38^. The surface of such prepared sample is observed in an SEM and images are taken for further analysis. Regarding hemocompatibility evaluation, attention is mainly focused on the size of the sample area covered by blood elements, their condition and morphology, and the presence of fibrin fibres. From the morphological changes of platelets (esp. formation of filopodia), it is possible to infer their activation^39^.

In the present work, the interaction of human blood with a polymeric surgical mesh has been studied by means of SEM. The procedure of sample fixation was optimized, reducing the use of toxic substances. SEM images of the surgical mesh with adhered blood elements were analysed in ImageJ software to determine the degree of surface coverage with blood cells. In addition, the blood elements’ arrangement on the surface and their condition were assessed qualitatively. The method proposed can be used to assess the thrombogenic potential of medical devices and their adhesiveness to blood cells.

## Materials and methods

### Sample preparation

For testing different fixation procedures, an MD was chosen, which showed moderate thrombogenic activity and blood elements adhesion, plus the formation of a fibrin network during the pilot study. It was a surgical mesh PP Mesh (VUP Medical, a.s., Czech Republic) made of polypropylene, commonly used in the healthcare industry for hernia plastic surgery.

Human whole blood was collected from healthy donors in the morning. The sampling was performed by a professionally qualified sampling nurse from an accredited laboratory. All donors signed written informed consent before the collection. All experimental protocols were approved by the Committee for Research Ethics at the University of Hradec Králové (statement No. 9/2021). All methods were carried out in accordance with relevant guidelines and regulations. Blood was collected into 4 ml VACUETTE® tubes (Dialab s.r.o., Slovak Republic) using a vacuum system. Immediately after collection, the blood was treated with an anticoagulant (heparin) and gently mixed by inverting the tube several times. Subsequently, a tested sample of the surgical mesh with a square size of 10 × 10 mm was inserted into the tube with blood. A triplicate sample was used for each variant of fixation for SEM. The blood was incubated together with the sample for 30 min at 37 °C in an Isotemp GPD05 water bath (Thermo Fisher Scientific, USA) with regular gentle stirring.

After incubation, the samples were carefully removed from the blood, gently rinsed with phosphate-buffered saline (PBS), and treated according to the planned fixation variants (see Table 1). The following chemicals were used to prepare the solutions: May–Grünwald eosin methylene blue solution (VWR, USA), 4% aqueous PFA (VWR, USA), absolute ethanol (VWR, USA), acetone (VWR, USA), 25% aqueous GA (Electron Microscopy Sciences, USA), 2% GA and 2% PFA in PBS (Electron Microscopy Sciences, USA), 3% GA in PBS (Electron Microscopy Sciences, USA), 3% GA in cacodylate buffer (Electron Microscopy Sciences, USA), PBS tablets (Carl Roth GmbH, Germany), and 4% osmium tetroxide (Carl Roth GmbH, Germany).

**Table 1:** Variants of fixation and preparation of a medical device sample after its incubation in blood.

Variant	1st chemical	Washing	2nd chemical	3rd chemical	Drying	Coating
1	100% May–Grünwald solution	PBS	Ethanol series	–	CPD	Pt 6 nm
2	100% May–Grünwald solution	PBS	Ethanol series	Acetone series	CPD	Pt 6 nm
3	50% May–Grünwald solution	PBS	Ethanol series	–	CPD	Au 6 nm
4	50% May–Grünwald solution	PBS	Acetone series	–	CPD	Au 7 nm
5	4% aqueous PFA	PBS	Ethanol series	–	CPD	Au 6 nm
6	4% aqueous PFA	PBS	OsO_4_, 1 h, RT	Ethanol series	CPD	Au 7 nm
7	Ethanol series	PBS	–	–	CPD	Au 6 nm
8	Acetone series	PBS	–	–	CPD	Au 7 nm
9	25% aqueous GA	PBS	Ethanol series	–	CPD	Au 6 nm
10	25% aqueous GA	PBS	OsO_4_, 24 h, 4 °C	Ethanol series	CPD	Au 6 nm
11	2% GA, 2% PFA in PBS	PBS	Ethanol series	–	CPD	Au 6 nm
12	2% GA, 2% PFA in PBS	PBS	OsO_4_, 24 h, 4 °C	Ethanol series	CPD	Pt 6 nm
13	3% GA in PBS	PBS	Ethanol series	–	CPD	Pt 6 nm
14	3% GA in PBS	PBS	OsO_4_, 24 h, 4 °C	Ethanol series	CPD	Pt 6 nm
15	3% GA in cacodylate solution	PBS	Ethanol series	–	CPD	Pt 6 nm
16	3% GA in cacodylate solution	PBS	OsO_4_, 24 h, 4 °C	Ethanol series	CPD	Pt 6 nm
17	25% aqueous GA	PBS	OsO_4_, 1 h, RT	Ethanol series	CPD	Au 7 nm
18	2% GA, 2% PFA in PBS	PBS	OsO_4_, 1 h, RT	Ethanol series	CPD	Au 7 nm
19	3% GA in PBS	PBS	OsO_4_, 1 h, RT	Ethanol series	CPD	Au 7 nm
20	3% GA in cacodylate solution	PBS	OsO_4_, 1 h, RT	Ethanol series	CPD	Au 7 nm
21	100% May–Grünwald solution	PBS	OsO_4_, 1 h, RT	Ethanol series	CPD	Au 7 nm
22	50% May–Grünwald solution	PBS	OsO_4_, 1 h, RT	Ethanol series	CPD	Au 7 nm
23	100% May–Grünwald solution	PBS	OsO_4_, 1 h, RT	Acetone series	CPD	Au 7 nm
24	50% May–Grünwald solution	PBS	OsO_4_, 1 h, RT	Acetone series	CPD	Au 7 nm
25	2% GA, 2% PFA in PBS	PBS	OsO_4_, 1 h, RT	Acetone series	CPD	Au 7 nm

The first chemical was left to act for 10 min each time, followed by a phosphate-buffered saline (PBS) rinse, followed by exposure to another series of chemicals. Ethanol dehydration series—gradually increasing ethanol concentration every 10 min (30, 40, 50, 60, 70, 80, 90, 96, and 100% ethanol), acetone series—gradually increasing acetone concentration every 10 min (30, 50, and 100% acetone). PFA—paraformaldehyde, GA—glutaraldehyde, OsO_4_—4% osmium tetroxide, RT—room temperature, CPD—slow drying of the sample using CO_2_.

After completing the dehydration in an ethanol or acetone series, the samples were dried in an EM CPD 300 critical point drying device (Leica, Germany). The CPD device chamber was filled with the same dehydration substance (ethanol or acetone), and the samples in a special CPD holder were inserted there. The CPD settings recommended by the manufacturer for human blood samples^40^ were used for all our samples (CO_2_ in: speed—slow, delay—120 s,exchange: speed—1, cycles—16; gas out: heat—slow, speed—slow 20%). The whole drying process took about 3 h.

The dried samples were stuck on aluminium pads with a conductive carbon tape. Then they were sputter-coated with a thin layer of metal. This procedure increases the samples’ electrical and thermal conductivity and also the yield of secondary electrons from their surface^14^, leading to a better quality of SEM images. The coating was carried out using an EM ACE 200 vacuum coater (Leica, Germany), working in a diffusion mode. Different types of metals (gold, platinum) with a coating thickness of 6–7 nm were tested (see Table 1).

### Imaging and qualitative evaluation of the samples

The mesh samples were imaged by a FlexSEM 1000 scanning electron microscope (Hitachi, Japan) operated in a secondary electrons mode at accelerating voltages of 10–15 kV. The magnification was set to 65×.

**Figure 1:**
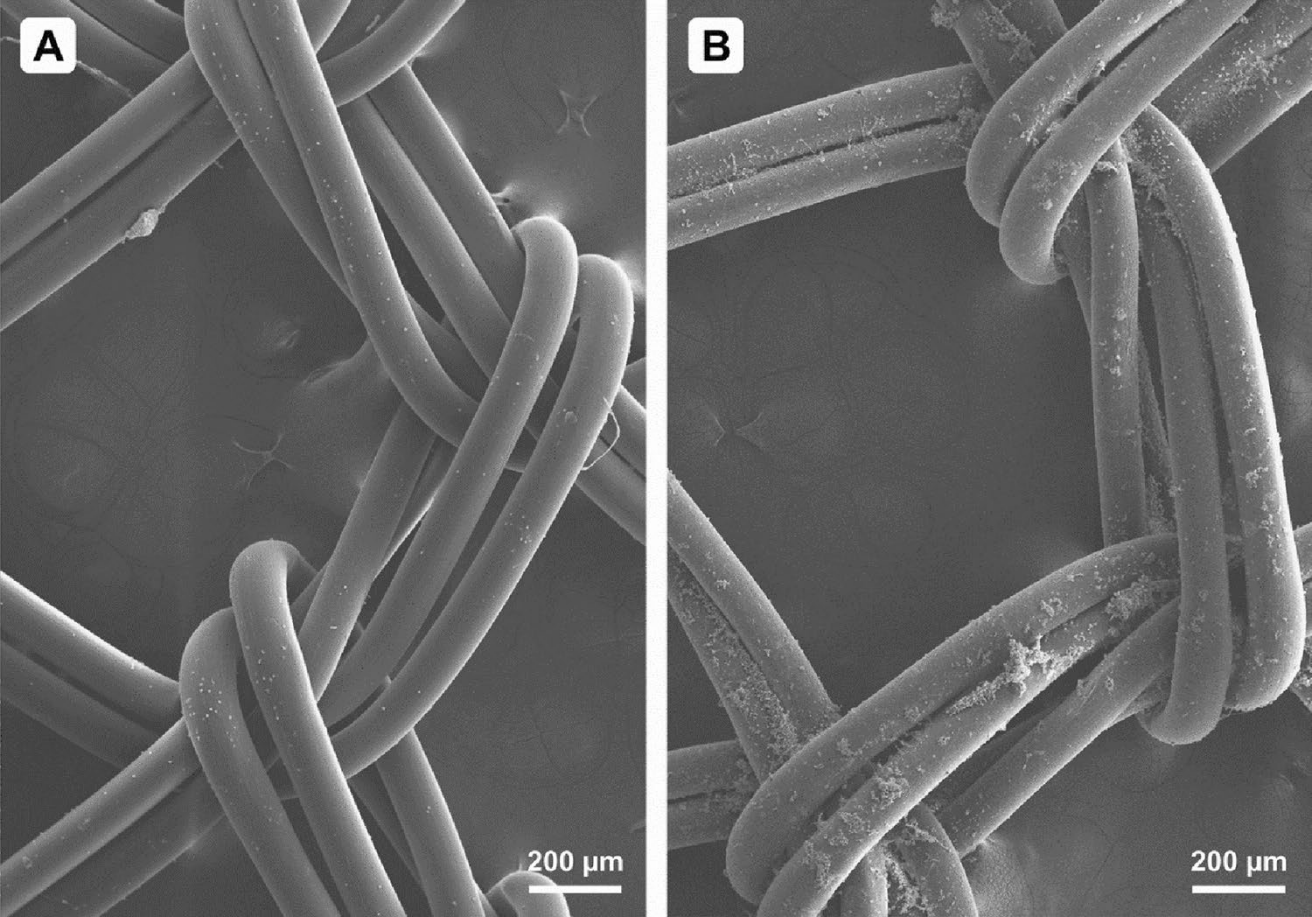
(**A**) A sample with a very low degree of blood cell adhesion on the surface—variant 22 from Table 1. Magnification 65× . Subjectively evaluated degree of adhesion: 0 (0–10% surface coverage), degree of adhesion calculated using ImageJ: 1.81%. (**B**) A sample with a high degree of blood cell adhesion on the surface—variant 1 from Table 1. Magnification 65×. Subjectively evaluated degree of adhesion: 3 (surface coverage > 50%), degree of adhesion calculated using ImageJ: 24.61%.

Images of randomly selected quadrangular structures consisting of individual mesh filaments were taken. Examples of such images are given in Fig. 1. The images were subsequently used to evaluate the degree of blood elements adhesion in ImageJ software, as described below.

This was followed by a gradual magnifying of the individual filament surfaces and an examination of the presence of various blood element types. The condition of the blood elements found was assessed, any non-physiological findings were recorded, and images were taken. All observations, such as the presence or absence of fibrin fibres and activated platelets and the overall state of the cells, were summarized in a table.

Furthermore, the subjective degree of surface coverage (degree of adhesion) was determined according to a scale of 0–3 (0: 0–10% coverage of the MD surface, 1: 10–25% coverage, 2: 25–50% coverage, 3: 50–100% coverage). Figure 1 points out the difference in the degree of adhesion for different variants of fixation.

### Quantification of the degree of adhesion using ImageJ

The degree of adhesion was assessed quantitatively from the SEM images taken for each sample at 65× magnification. For this purpose, the image processing and analysis software ImageJ 1.53 k (National Institutes of Health, USA; available for free at https://imagej.net/ij/) was used. After opening the image in ImageJ, the correct scale was initially set by assigning the corresponding number of pixels to the length of the scale bar displayed in the image.

**Figure 2:**
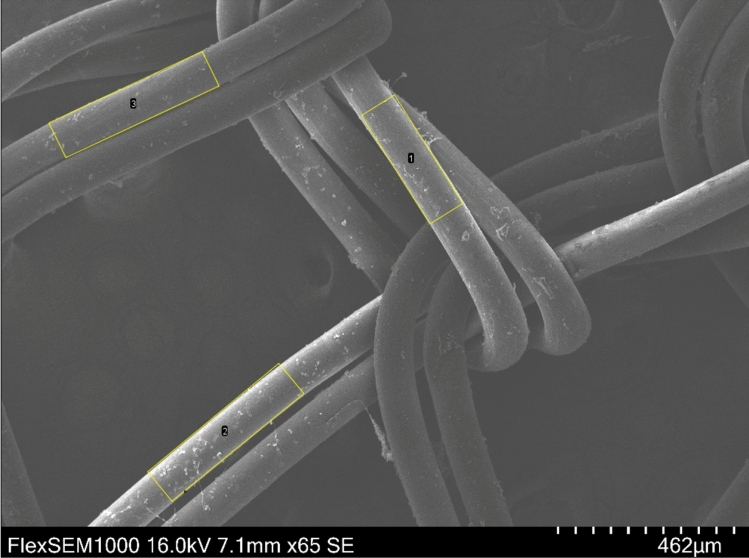
Positioning of rectangles delimiting areas for measuring the degree of blood elements’ adhesion on the surface of a medical device (surgical mesh). Sample preparation: variant 9 from Table 1. Magnification 65×.

Subsequently, three identical rectangles of precisely defined dimensions (450 × 100 μm) were randomly positioned on the MD in the image to delimit the measurement areas (see Fig. 2). The individual cells (or their clusters) located in these areas were then measured: Each object was demarcated manually along its perimeter and its area was determined using the ImageJ function “Measure”, which calculates the area of selection from the number of square pixels contained inside and the set scale. The resulting values were expressed in μm and rounded to 2 decimal places. If a cell was located on the bounding rectangle’s perimeter, only its part lying inside the rectangle was demarcated and included in the calculation. After determining the areas of all objects inside one rectangle, the results were saved and the analysis continued the same way for the remaining two bounding rectangles. When finished, the image with all the objects marked was saved in the TIF format.

The obtained data were then statistically processed. For each sample, the data for each bounding rectangle were processed separately. Also, data aggregated from the triplicates were evaluated. The total coverage of the surface with cells and their clusters together was calculated, as well as the proportion of individual cells in the covered surface and the proportion of clusters in the covered surface. Objects with an area < 40 μm^2^ were considered as single cells and objects above 40 μm^2^ as clusters (using a formula “COUNTIF”). The degree of adhesion was determined as the total covered area within the three rectangles divided by the total area of these rectangles.

### Testing other sample types

After selecting the best methods of fixation, other types of MD, for which we assumed positive and negative hemocompatibility, were tested to verify the proposed fixation methods. The first control MD was a Tachosil haemostatic fibrin-based surgical patch (Baxter International Inc., USA), which is used to stop bleeding during surgical procedures. We therefore expected a strong adhesive reaction of its surface with blood and, at the same time, the activation of blood elements associated with coagulation. The second control MD was a knitted vascular prosthesis (VUP Medical, a.s., Czech Republic), which was expected to have good hemocompatibility and thus a low percentage of adhesion and coagulation. These samples were tested according to the established protocol described in this article with fixation variants 1 and 25.

## Results

### Degree of adhesion

The highest degree of adhesion analysed using the ImageJ software was found for fixation variant 1, i.e., for samples fixed in 100% May–Grünwald solution, followed by gradual transfer to absolute ethanol, drying in a CPD, and coating with a 6 nm layer of platinum. The degree of adhesion was determined to be 24.6%, and the proportion of cell clusters was about 22% in these samples.

On the contrary, the lowest degree of adhesion (1.1%) was determined for variant 6, in which fixation was carried out using 4% PFA, followed by exposure to osmium tetroxide for 1 h and gradual transfer to absolute ethanol. The proportion of cell clusters was approx. 8% in this variant.

**Figure 3:**
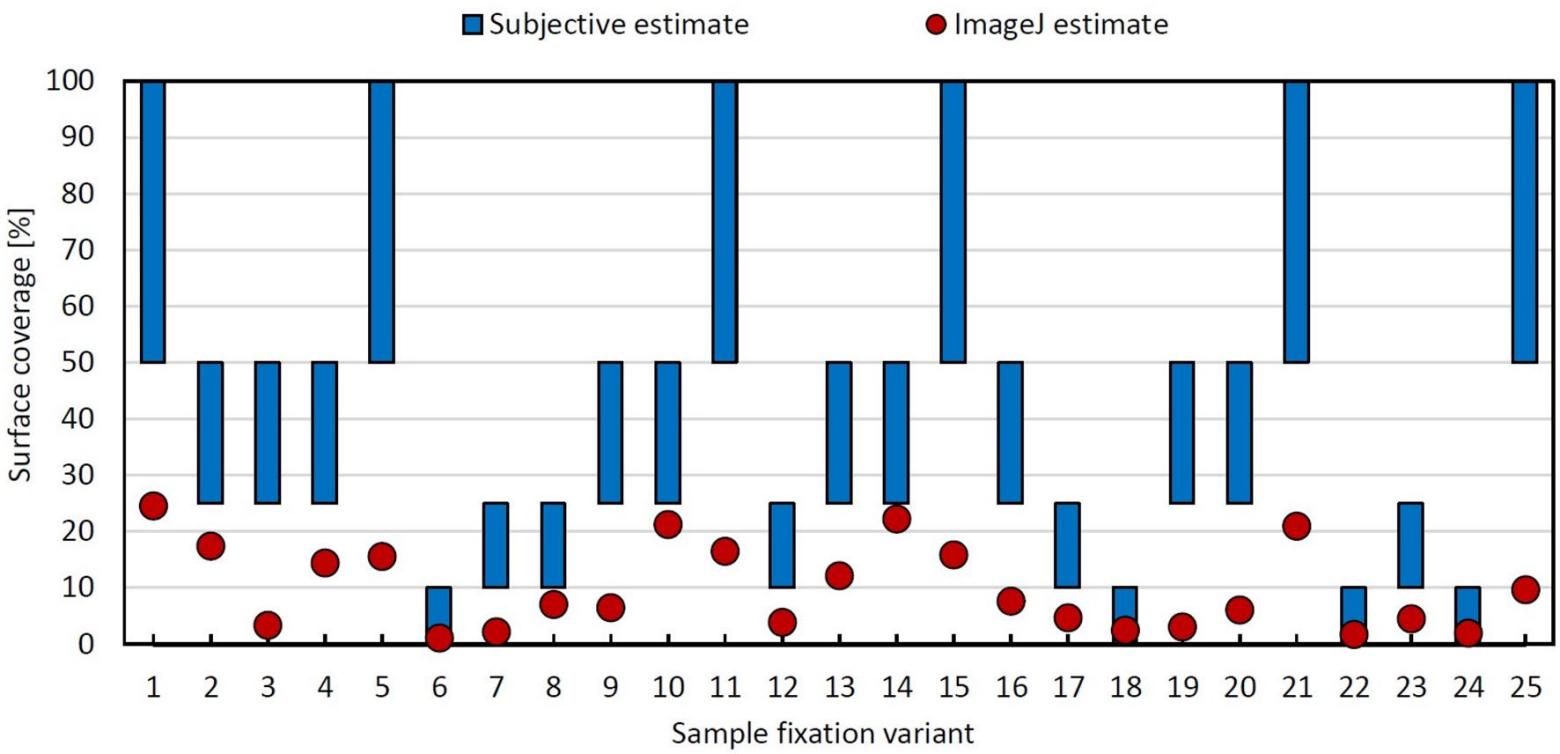
Subjectively assessed adhesion of blood elements on the medical device surface compared with quantitative results from ImageJ.

In Fig. 3, surface coverage calculated in ImageJ is compared with the results of subjective coverage evaluation for all tested variants. The results clearly show that the subjective estimate of the degree of adhesion is significantly above the values determined using ImageJ.

### Formation of cell clusters

**Figure 4:**
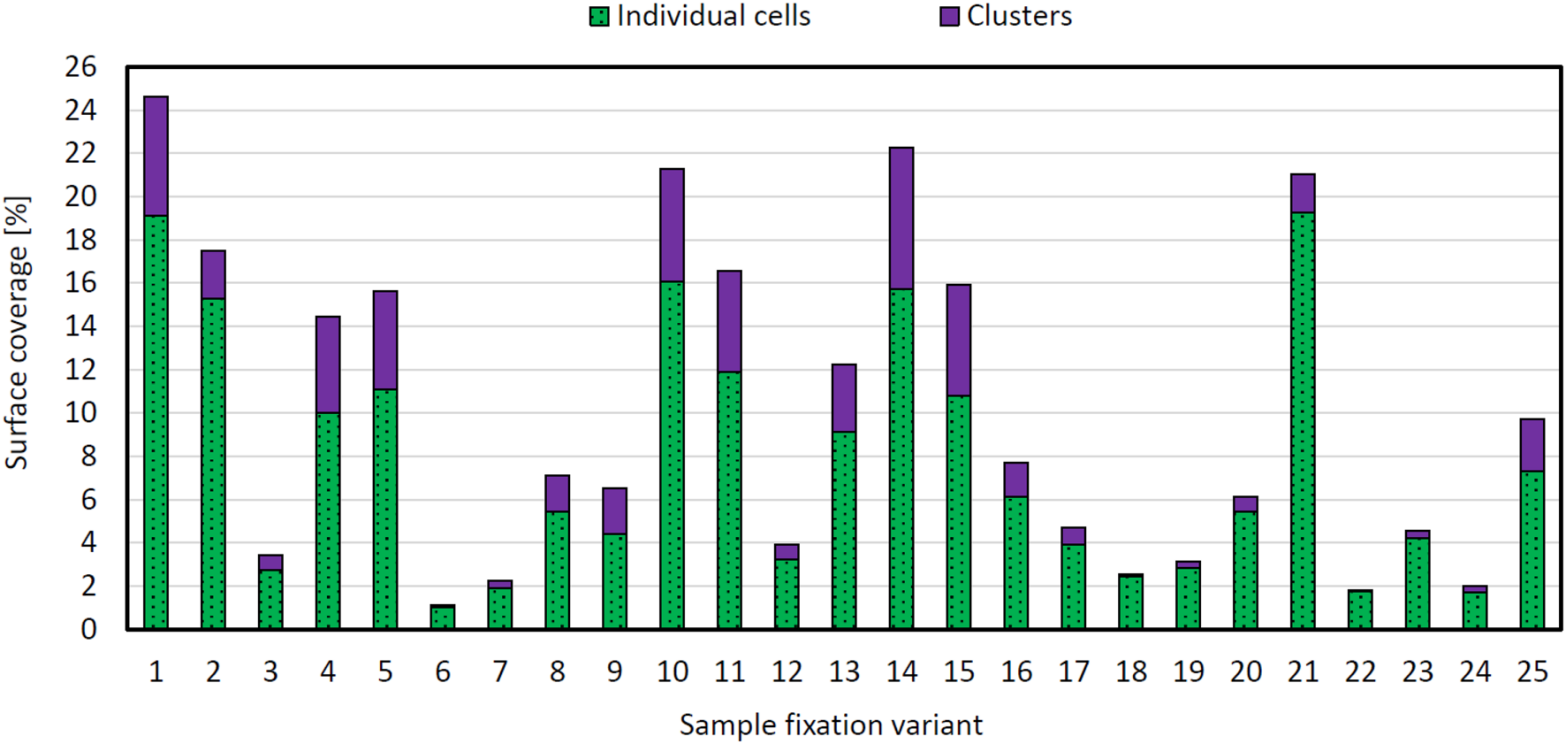
The proportions of individual cells (area < 40 μm^2^) and their clusters (area > 40 μm^2^) on the medical device surface. Measured in three randomly located 45,000 μm^2^ rectangles on the material surface. The displayed values were determined from SEM images using ImageJ software.

Proportions of individual cells versus cell clusters on the MD surfaces are shown in Fig. 4. Individual cells occupy a larger surface area on all samples, but there is considerable variation in the relative abundance of cells and clusters among the fixation variants. The highest tendency to form clusters on the MD surface was found for variants 9 (32.2%) and 15 (32.1%). In both cases, glutaraldehyde was used for fixation. The lowest proportion of clusters (2.9%) was detected in variant 22, in which 50% May–Grünwald solution and OsO_4_ were used.

### Evaluation of specimen fixation

**Figure 5:**
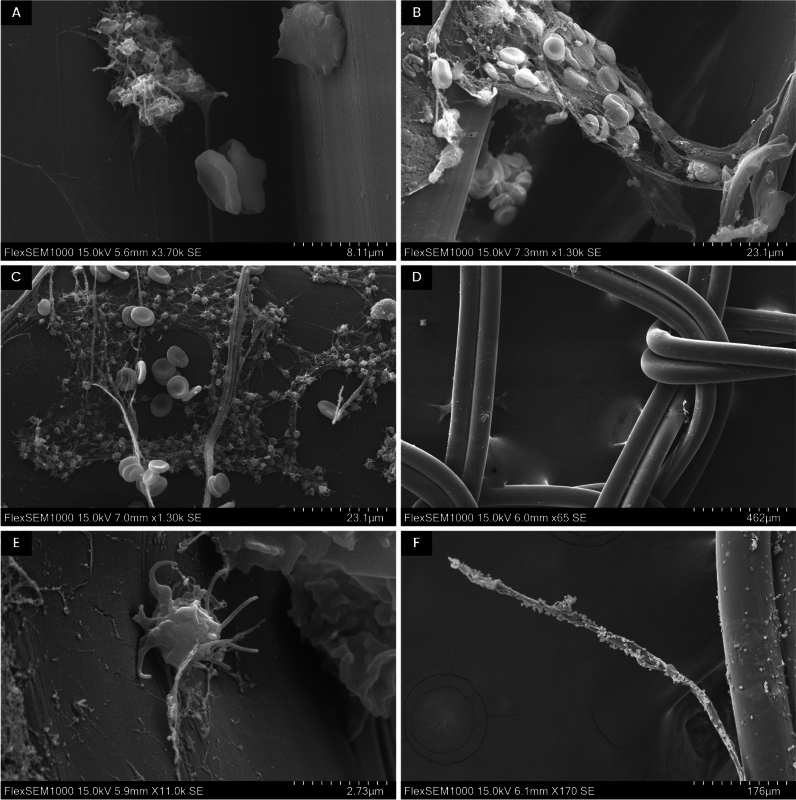
Biological structures attached to the medical device surface. (**A**) A cluster of activated platelets (left) and deformed erythrocytes (right) in a sample fixed according to variant 6, magnification 3700×. (**B**) A network of fibrin fibres fixed between sections of a surgical mesh with attached erythrocytes and platelets (variant 11), magnification 1300×. (**C**) Fibrin fibres with erythrocytes and platelets on the surface of a surgical mesh (variant 15), magnification 1300×. (**D**) Filaments of a surgical mesh (variant 18), magnification 65×. (**E**) An activated platelet with prominent filopodia on the surface of a surgical mesh (variant 25), magnification 11,000×. (**F**) A fibrin fibre attached to the mesh surface (variant 25), magnification 170×.

The evaluation of the appearance of the MD surfaces after the incubation is summarized in Table 2, which contains information on the presence/absence of activated platelets and fibrin fibres, the overall state of the cells, and other findings related to the blood elements observed. Examples of related SEM images are shown in Fig. 5.

**Table 2:** Evaluation of the appearance of cells attached to the surgical mesh surface.

Variant	Overall cell condition	Surface coverage	Platelet activation	Fibrin fibres	Other observations
1	Very good condition, single cells	3	YES	YES	Bacteria on the surface, slightly swollen erythrocytes
2	Compact mass, plates, unrecognizable blood elements	2	NO	NO	Bacteria on the surface, slightly swollen erythrocytes, most of the cells form undefinable clumps that detach from the mesh in the form of sheets (probably due to acetone)
3	Very good condition	2	FEW	YES	–
4	Some cells are single, others clustered into plates	2	YES	FEW	The cells partially protrude into space, they cluster more into plates that tend to detach; deformation of erythrocytes, apparently covered with bacteria
5	Excellent cell condition, fixed even in space	3	FEW	YES	Fibrin fibres also protrude into space, very well fixed
6	Non-physiological state of cells, small number of cells	0	YES	NO	Almost nothing on the surface, cell lysis, but beautiful activated clusters of platelets (Fig. 5A)
7	Deformation, less cell adhesion	1	NO	NO	Cell deformation, clusters
8	Very poor cell condition	1	NO	NO	Very few cells, deformed cells, unidentifiable blood elements clumped into plaques
9	Physiological state of cells, little adhesion	2	NO	FEW	Undeformed cells, but in a smaller amount (probably too strong glutaraldehyde)
10	Slight deformation of cells, clusters	2	NO	FEW	Clusters of cells apparently slightly damaged during fixation (too strong glutaraldehyde and subsequent action of osmium tetroxide), only one nice lymphocyte found
11	Physiological state of cells	3	FEW	YES	In some places erythrocytes stacked on top of each other, without deformations; fibrin fibres with trapped erythrocytes hold even in space (Fig. 5B)
12	Deformed cells	1	NO	FEW	Cells only in a few places, little coverage, rather deformed surface, degradation of the mesh filament (cracks), short fibrin fibres
13	Good cell condition	2	YES	YES	Undifferentiated sheets of cells, but also individual cells
14	Physiological state of cells, individual blood elements	2	YES	YES	Few clumps, rather individual cells, beautiful fibrin networks
15	Physiological state of cells, cells individually and in clusters	3	YES	YES	Cells individually and in clusters, beautiful activated platelets (Fig. 5C), fibrin fibres protruding into space
16	Little adhesion, cell deformation	2	NO	NO	Deformed cells and only in small numbers, no activated platelets, compared to sample 15 (without osmium)
17	Good condition of most cells, but some cells deformed; little adhesion	1	FEW	FEW	Little surface coverage, rather individual erythrocytes
18	Poor cell condition, clusters	0	NO	YES	Very few cells on the surface, clots of indeterminate structure found, almost no individual cells (Fig. 5D)
19	Physiological state of cells, cells individually and in clusters	2	YES	NO	More cells compared with variant 18, activated platelets individually and in clusters
20	Physiological state of cells	2	YES	YES	Slightly larger surface covered (compared with variant 19), larger clumps, fibrin fibres (but not protruding into space), nice activated platelets
21	Good cell condition, high degree of adhesion	3	YES	YES	Very much cells on the surface and in space, bacteria
22	Non-physiological state of cells	0	YES	NO	Very few cells (rather individually), round erythrocytes, cell deformation
23	Some cells with a disrupted membrane	1	YES	NO	Very few cells, but up close the cells look a bit better than in variant 22, the sample was charging
24	Average cell condition, little adhesion	0	NO	FEW	Swollen surgical mesh, very few cells
25	Very good physiological state of cells, high degree of adhesion; cells individually, in clusters, and in space too	3	YES	YES	Large surface coverage, erythrocytes individually and in clusters, erythrocytes of the classic double-concave shape, platelets with stalks; no bacteria; activated platelets and lymphocytes (Fig. 5E), fibrin with trapped erythrocytes even protruding into space (Fig. 5F)

The presence of activated platelets and fibrin fibres was monitored. The physiological state of blood elements, especially erythrocytes, was assessed too. Surface coverage was assessed subjectively based on a scale of 0–3 (0: 0–10% coverage of the medical device surface, 1: 10–25% coverage, 2: 25–50% coverage, 3: 50–100% coverage).

The best condition of the cells, including an extensive solid fibrin network, was achieved with variant 25, i.e. fixation using 2% GA and PFA in PBS, followed by a 1-h-long exposure to osmium tetroxide. At first glance, a high MD coverage—compared with the other fixation variants—could be noticed. The erythrocytes did not exhibit any deformations of their classic biconvex shape and were located both individually and in clusters. The activated platelets had firmly fixed stems without visible damage. There were no bacteria on the surface. In addition to the activated platelets (Fig. 5E), also lymphocytes were observed. Fibrin fibres with trapped erythrocytes were also fixed in free space (Fig. 5F).

Variant 15 also showed very good results and a physiological state of the cells. Activated platelets were found in large numbers, and strengthened fibrin networks raised even into free space. The degree of adhesion was subjectively assessed at 3 (the highest value of the scale, > 50%). Some parts of the MD surface in this sample were covered by networks of activated platelets and fibrin fibres with attached erythrocytes (Fig. 5C). It is worth noting that these samples were fixed without the use of osmium tetroxide.

Other successful types of fixation included variants 1, 3, 5, 11, 13, 14, and 19–21. For example, in variant 11 (2% GA, 2% PFA, and ethanol series), cells were in very good condition and undeformed. In some places, erythrocytes were stacked on top of each other, and fibrin fibres extended into free space. Erythrocytes and other blood elements were also trapped in these fibrin networks (Fig. 5B).

On the contrary, the worst results were obtained for variants 2, 6, 7, 8, 16, 18, and 22. Variant 6 (4% PFA, OsO_4_ for 1 h, ethanol series) resulted in a non-physiological state of cells, which were deformed and largely lysed. Generally, almost no blood elements were attached to the MD surface. An exception was the finding of a few clusters of activated platelets (Fig. 5A).

Very few cells attached to the surface and some precipitates of indeterminate structures were found in variant 18 (2% GA, 2% PFA in PBS, OsO_4_ for 1 h, ethanol series) —see Fig. 5D. In some cases, formations resembling bacteria in appearance and size appeared on the MD surface, or rather on the surface of blood elements (variants 1, 2, 4, 21).

Variants 9, 10, 17, 23, and 24 were characterized by average results, with usually lower cell adhesion and slightly deformed cells.

### Evaluation of other MD types

**Figure 6:**
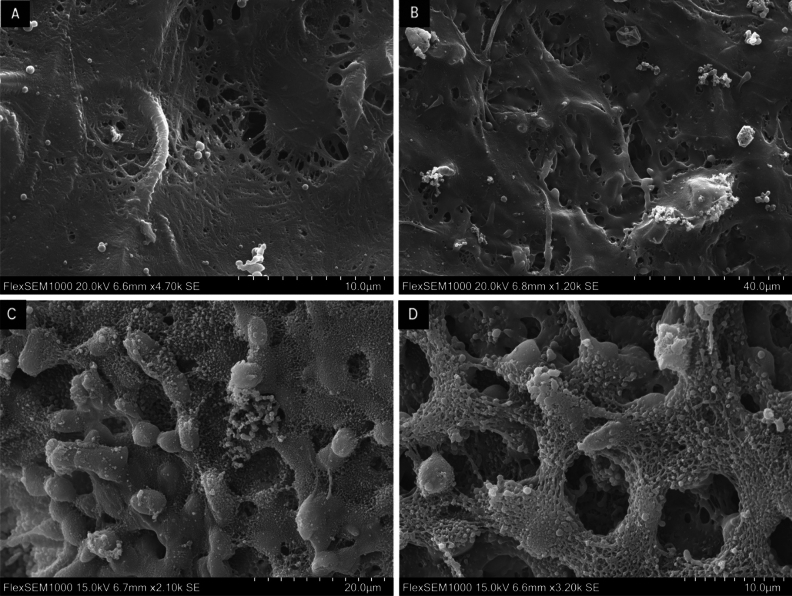
Biological structures attached to the medical device surface. (**A**) The surface of the vascular prosthesis fixed by variant 1 (100% May–Grünwald solution), magnification 4700×. (**B**) The surface of the vascular prosthesis fixed by variant 25 (2% glutaraldehyde and paraformaldehyde), magnification 1200×. (**C**) The surface of Tachosil haemostatic fibrin-based surgical patch fixed by variant 1, magnification 2100×. (**D**) The surface of Tachosil haemostatic fibrin-based surgical patch fixed by variant 25, magnification 3200×.

The results for the control samples were as follows: The vascular prosthesis fixed with variant 1 achieved only 3.8% degree of adhesion, with single cells (98.2%) predominating over the presence of clusters (1.8%). With variant 25, the degree of adhesion was 6.0% and only a low incidence of cell clusters (5.2%) was observed too. In both variants of the vascular prosthesis sample, most of the cells were in a physiological state, and no activated platelets were found. On the contrary, Tachosil fixed with variant 1 reached an 80.9% degree of adhesion and cell clusters predominated here (98.9%). With variant 25, the degree of adhesion for Tachosil was 76.5%, the proportion of cell clusters being 99%. In both of these variants, most of the cells were disrupted and a large number of activated blood elements (mainly platelets) and fibrin fibres were found. SEM images of the control samples are shown in Fig. 6.

## Discussion

This research aimed to determine the most suitable type of fixation of blood cells attached to the surface of a medical device after its interaction with blood and to create a methodology for evaluating the degree of blood adhesion using SEM.

Testing the fixation variants identified as the best (variants 1 and 25) was carried out on the control medical devices—a vascular prosthesis (as an MD with low thrombogenic activity) and a haemostatic fibrin-based surgical patch (as an MD with high thrombogenic activity and blood element adhesion). This testing confirmed the positive fixation results obtained with the surgical mesh. The assumed thrombogenic activity of these samples was confirmed using both fixation variants. No significant differences were found between the fixation with May–Grünwald solution and glutaraldehyde. Only in the vascular prosthesis fixed with variant 25, a slightly higher amount of cell clusters and degree of adhesion compared with variant 1 were found.

Different alternative methods can also be used to determine cell adhesion. One of them is a single-cell force spectroscopy (SCFS). The advantage of this method lies in the possibility of studying living cells under almost physiological conditions. This is a substantial difference from SEM measurements, where the samples have to be fixed first. In addition, SCFS makes it possible to study the properties of individual adhesion-receptor-ligand interactions^45,46^. On the other hand, the SCFS method is time-consuming because each cell is tested individually. In addition, experiments with long contact times are disadvantaged by thermal drift^46^.

To determine platelet activation, platelet aggregometry could be employed. However, it is insufficient to evaluate the adhesion of blood elements (not only platelets) on the MD surface. This is because this method works with either whole blood or plasma, so it cannot be used directly to analyse blood adhered to the sample surface^47^.

Another option is fluorescence microscopy, but imaging cell adhesion by a fluorescence microscope is limited by its achievable spatial resolution. Further, a confocal microscope can be used to observe the surface of a sample that is too thick for a classic optical microscope^48^. But, except for a few special cases, biomolecules in cells are not naturally fluorescent, so it is usually necessary to manipulate the samples in a certain way to make their parts fluoresce. For platelets, for example, mouse anti-human CD62P monoclonal antibodies are used to enhance fluorescence^45^. A drawback of such an approach is relatively time-consuming sample preparation involving a considerable number of steps.

A great advantage of SEM is its high resolution and the possibility of observing even very small objects with fine structure. Detailed topographical imaging, together with a high depth of field, is very beneficial for the evaluation of blood cell morphology and thus the changes associated with blood cell activation. On the contrary, the preparation of biological samples for SEM is quite complicated and requires special laboratory equipment, such as a critical-point dryer (CPD). The SEM measurement is carried out in a vacuum, which excludes observations of living organisms or liquid samples. The high price of the electron microscope can be a problem too.

Since toxic substances such as osmium tetroxide are commonly used for the fixation of biological samples for SEM measurements^14,27^, we made an effort to reduce the use of highly toxic substances to a minimum and to shorten the individual fixation steps. Emphasis was also placed on an effective and comprehensible evaluation of the obtained data. The goal was to create a clear and objective assessment method that goes beyond a mere subjective evaluation of the images obtained—as subjective methods can be highly inaccurate and misleading. For this purpose, ImageJ software was used, and a methodology for the measurement and quantitative evaluation of the obtained data was created.

As a part of this research, various substances with different concentrations have been combined and tested, such as the commonly used glutaraldehyde and paraformaldehyde in cacodylate buffer, in PBS or as an aqueous solution. Newly, May–Grünwald solution was also used for the sample fixation and assessment of the adhesion degree using an SEM. A significant advantage of the May–Grünwald solution is its lower toxicity compared with other fixative chemicals^41^.

Very good results of sample fixation were achieved in particular with the solution of 2% GA and 2% PFA in PBS (variants 11 and 25). In these samples, the state of the blood cells was physiological, the degree of adhesion was high, and fibrin fibres were firmly fixed not only on the MD surface but also in free space. Firm fixation of elements extending from the surface is especially important for samples with a formed fibrin network protruding above the surface and for the subsequent correct interpretation of the thrombogenicity of the tested sample.

A significant difference between variants 11 and 25 was the use of osmium tetroxide, which was absent in variant 11. Nevertheless, the results were very good and comparable. The degree of adhesion was even higher in variant 11. These results demonstrate the possibility of excluding toxic OsO_4_ from the fixation procedure.

The same was confirmed by the results of fixation with 3% GA in cacodylate buffer (variant 15 without OsO_4_, variants 16 and 19 with OsO_4_). In the variant without osmium tetroxide, the highest degree of adhesion was determined, cells were in a physiological state and fixed even in free space. On the contrary, cells in variant 16 were deformed and attached only in undefined clusters on small parts of the MD surface. These samples had been exposed to OsO_4_ for 24 h, unlike in variant 19, where the exposure to OsO_4_ lasted only 1 h. The shorter exposure to osmium tetroxide led to better cell condition and fixation in free space compared with the longer fixation time. Not only from these results, it follows that if we decide to use osmium tetroxide, its action should be restricted to maximally 1 h.

No clear differences were found between PBS and cacodyl buffers utilization. In some cases, cacodylate buffer appeared to give better results. This is evidenced, for example, by the differences between the samples fixed in 3% GA in PBS (variant 13) and in cacodylate buffer (variant 15)—greater adhesion and better cell condition were exhibited by variant 15. In other cases, however, the results were the opposite. Thus, it is not possible to unequivocally determine which of the given buffers is better. Both cacodylate buffer and PBS can be used, but considering the higher toxicity of cacodylate buffer^42,43^, PBS appears to be preferable. In any case, using either of these buffers is better than using purely aqueous solutions, which do not provide the cells with physiological conditions.

Based on our results, a 25% aqueous solution of GA cannot be recommended for fixing blood samples and determining the adhesion degree. Such concentrated solution essentially washed away individual cells from the MD surface, and mostly only larger clusters of cells remained there. In addition, some cells got deformed. Similar results were obtained without dependence on the additional fixation steps (without OsO_4_, OsO_4_ for 1 h, OsO_4_ for 24 h), showing that the reason was a too high concentration of the solution and the absence of buffer.

Results regarding the applicability of the May–Grünwald solution, which is ordinarily used to fix blood cells^41^ but not commonly used for evaluating thrombogenicity and degree of adhesion by an SEM, have been diverse. Fixation with a 100% solution was better than fixation with a 50% solution. A disadvantage of the May–Grünwald solution is the need for a higher level of sterility compared with other variants due to its susceptibility to infections. Bacteria were noted on the surface of some samples.

When this solution was used in combination with acetone, the fixed cells usually clumped into unidentifiable plaques that peeled off from the MD surface. Acetone was, therefore, found to be completely inappropriate. A suitable combination that produced highly promising results was the use of 100% May–Grünwald solution in SEM purity and subsequent conversion to absolute ethanol (variant 1), optionally including 1 h of OsO_4_ exposure (variant 21). In both these cases, the highest degrees of adhesion were achieved, and the cells were without significant signs of pathophysiological action.

In general, using ethanol or acetone series alone without prior prefixation and strengthening of cell structures with other substances is completely insufficient. It was confirmed by the results obtained for all samples fixed according to variants 7 and 8: acetone or ethanol essentially washed the unfixed cells from the MD surface and the resulting degree of adhesion was very low. In addition, the cells were very deformed. So, it is always necessary to fix the samples properly with stronger fixatives before drying, no matter how gentle the CPD procedure is. The importance of fixatives is that they strengthen the cells and stabilize their protein structures^11,15,24^.

The last variations on the sample preparation consisted in surface coating: gold and platinum with layer thicknesses of 6 and 7 nm were tested. Since sputtered gold forms larger grains than platinum, the use of Pt is preferred to achieve a better resolution of an SEM^44^. Our aim was, therefore, to find out whether the choice of coating material would affect the topography of our samples observed by SEM. However, no significant differences were noted between the Au and Pt sputter coating. With respect to the size of the observed blood elements, both Au and Pt provide image resolution sufficient for our research method. The coating layer thickness also did not affect the image quality. Considering the higher price of Pt, we recommend coating with a 6 nm layer of Au.

The results show substantial differences in the degree of blood cell adhesion as well as in the proportion of cell clusters formed, depending on the fixation variant used (see Fig. 4). We assume that different fixation variants do not directly affect the adhesion, but better fixation methods stabilize the cells enough so that the subsequent steps, esp. washing and drying, do not remove them from the surface. It subsequently affects the determined degree of adhesion. This may be especially important for samples with moderate thrombogenic activity, which contain fewer fibrin fibres that hold the surrounding blood elements on the MD surface even during the sample handling. The formation of cell clusters could possibly be influenced by the reaction with the fixative at the beginning of its action, if the fixative is not sufficiently strong and effective.

## Conclusions

We have demonstrated that SEM imaging can be conveniently used to evaluate the degree of blood adhesion on medical devices, as well as to study the interaction of blood with an MD in detail. As a part of this research, an appropriate sample preparation procedure has been proposed, which allows SEM observations of blood elements adhered on an MD surface. Glutaraldehyde and May–Grünwald solution were identified as the most suitable for the fixation of blood elements attached to the MD surface. The best results were achieved with the May–Grünwald solution, which has not been commonly used for these purposes yet. Specifically, it was variant 1, i.e. fixation using a 100% May–Grünwald solution and subsequent dehydration in an ethanol series, drying in a CPD and coating with platinum. Very good sample fixation results were also achieved with a solution of 2% GA and 2% PFA in PBS (esp. variant 25).

Our preparation procedure ensures not only the safe fastening of blood elements adhered to the MD surface, but also the firm fixation of structures protruding from the surface into free space, thereby more thoroughly capturing the result of the blood-MD interaction. Moreover, the use of highly toxic osmium tetroxide can be avoided—1-h postfixation with OsO_4_ is possible, but not necessary.

The degree of blood elements’ adhesion can be evaluated quantitatively using the newly created methodology based on the processing of SEM images with ImageJ software. Our proposed approach replaces the subjective assessment of surface coverage—which relies on mere qualitative observation of images—with a better-defined procedure, thus offering more precise and reliable results.

The described method can be especially useful when assessing the hemocompatibility of medical devices falling into the category in which the degree of thrombotic activity caused by blood interaction with foreign material has to be evaluated. Our introduced approach thus contributes to the important field of biocompatibility assessment of medical devices.

## Data Availability

All data generated or analysed during this study are included in this published article.
